# Explainable machine learning framework for cataracts recognition using visual features

**DOI:** 10.1186/s42492-024-00183-6

**Published:** 2025-01-17

**Authors:** Xiao Wu, Lingxi Hu, Zunjie Xiao, Xiaoqing Zhang, Risa Higashita, Jiang Liu

**Affiliations:** 1 Research Institute of Trustworthy Autonomous Systems and Department of Computer Science and Engineering, Southern University of Science and Technology, Shenzhen, 518055 Guangdong China; 2 School of Computer Science, University of Birmingham, Birmingham, B15 2TT United Kingdom; 3 Center for High Performance Computing and Shenzhen Key Laboratory of Intelligent Bioinformatics, Shenzhen Institute of Advanced Technology, Chinese Academy of Sciences, Shenzhen, 518055 Guangdong China; 4 Tomey Corporation, Nagoya, 4510051 Japan; 5 School of Computer Science, University of Nottingham Ningbo China, Ningbo, 315100 Zhejiang China; 6 Changchun University, Changchun, 130022 Jilin China

**Keywords:** Nuclear cataract, Anterior segment optical coherence tomography, Machine learning, Explainable, Visual feature

## Abstract

Cataract is the leading ocular disease of blindness and visual impairment globally. Deep neural networks (DNNs) have achieved promising cataracts recognition performance based on anterior segment optical coherence tomography (AS-OCT) images; however, they have poor explanations, limiting their clinical applications. In contrast, visual features extracted from original AS-OCT images and their transform forms (e.g., AS-OCT-based histograms) have good explanations but have not been fully exploited. Motivated by these observations, an explainable machine learning framework to recognize cataracts severity levels automatically using AS-OCT images was proposed, consisting of three stages: visual feature extraction, feature importance explanation and selection, and recognition. First, the intensity histogram and intensity-based statistical methods are applied to extract visual features from original AS-OCT images and AS-OCT-based histograms. Subsequently, the SHapley Additive exPlanations and Pearson correlation coefficient methods are applied to analyze the feature importance and select significant visual features. Finally, an ensemble multi-class ridge regression method is applied to recognize the cataracts severity levels based on the selected visual features. Experiments on a clinical AS-OCT-NC dataset demonstrate that the proposed framework not only achieves competitive performance through comparisons with DNNs, but also has a good explanation ability, meeting the requirements of clinical diagnostic practice.

## Introduction

Cataract is the leading disease of blindness and visual impairment globally. With global aging, the number of patients with cataracts will increase rapidly [1, 2] due to it being an age-related disease. Early intervention and cataracts surgery are two efficient approaches to improve vision and quality of life in patients with cataracts, as well as to reduce the social burden. The clinical manifestation of cataracts is a decrease in crystalline lens transparency, which is mainly caused by protein clumping. Depending on the opacity location, cataracts can be divided into three types: nuclear cataract (NC), cortical cataract (CC), and posterior subcapsular cataract (PSC) [3]. NC is a common type, and its clinical manifestations include gradual hardening and clouding in the nucleus region [4].

In recent years, anterior segment optical coherence tomography (AS-OCT) images have gradually been applied in the diagnosis and scientific research of ocular diseases, such as glaucoma [5–7] and cataracts [8, 9]. In terms of clinical cataracts diagnosis, studies have shown the existence of strong correlations between clinical features (e.g., the mean intensity value) and severity levels of NC [10, 11], providing a clinical basis for automatic NC classification. Zhang et al. [12] first applied the deep GraNet to address the NC classification task based on AS-OCT images. Xiao et al. [13] proposed a gated channel attention network (GCANet) for NC classification. Zhang et al. [9] designed an adaptive feature compression network for the automatic recognition of cataracts severity levels. Gu et al. [14] developed a ranking-based multi-scale feature calibration network for NC recognition. Although these deep neural networks (DNNs) have achieved good results on AS-OCT-based cataracts classification tasks, their explanations are poor in the decision-making process owing to their “black box” properties.

**Fig. 1:**
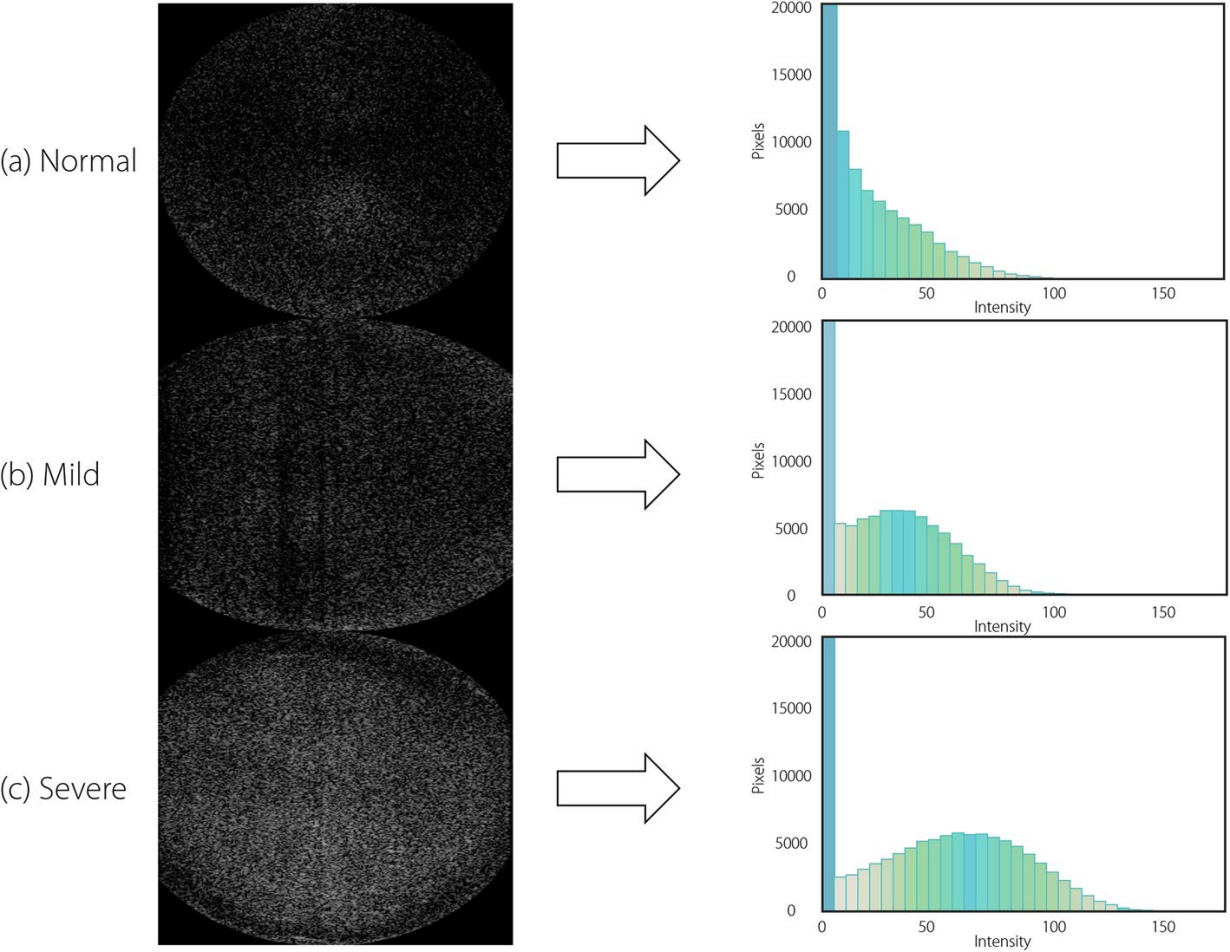
Three representative AS-OCT images and their corresponding AS-OCT-based histograms for three NC severity levels: **a** normal, **b** mild, and **c** severe. The AS-OCT-based histograms are built by counting the pixel values in the AS-OCT images. The pixel numbers of each interval are also referred to as intensity. In comparison, the original AS-OCT images of different NC severity levels are very similar, but their AS-OCT based histograms are significantly different

Moreover, previous works rarely extracted visual features from AS-OCT images and their transform variants for automated cataracts recognition, especially AS-OCT-based histograms, which have excellent explanations. Figure 1 (left) provides three representative AS-OCT images of three NC severity levels: normal, mild, and severe; it can be observed that the opacity differences among different NC severity levels are inapparent. Surprisingly, when the original AS-OCT images are transformed into AS-OCT-based histograms (Fig. 1 (right)), significant vision differences are observed among the NC severity levels via AS-OCT-based histograms visually. According to an extensive survey, existing methods have not systematically exploited the visual features extracted from AS-OCT-based histograms for explainable cataracts recognition.

**Fig. 2:**
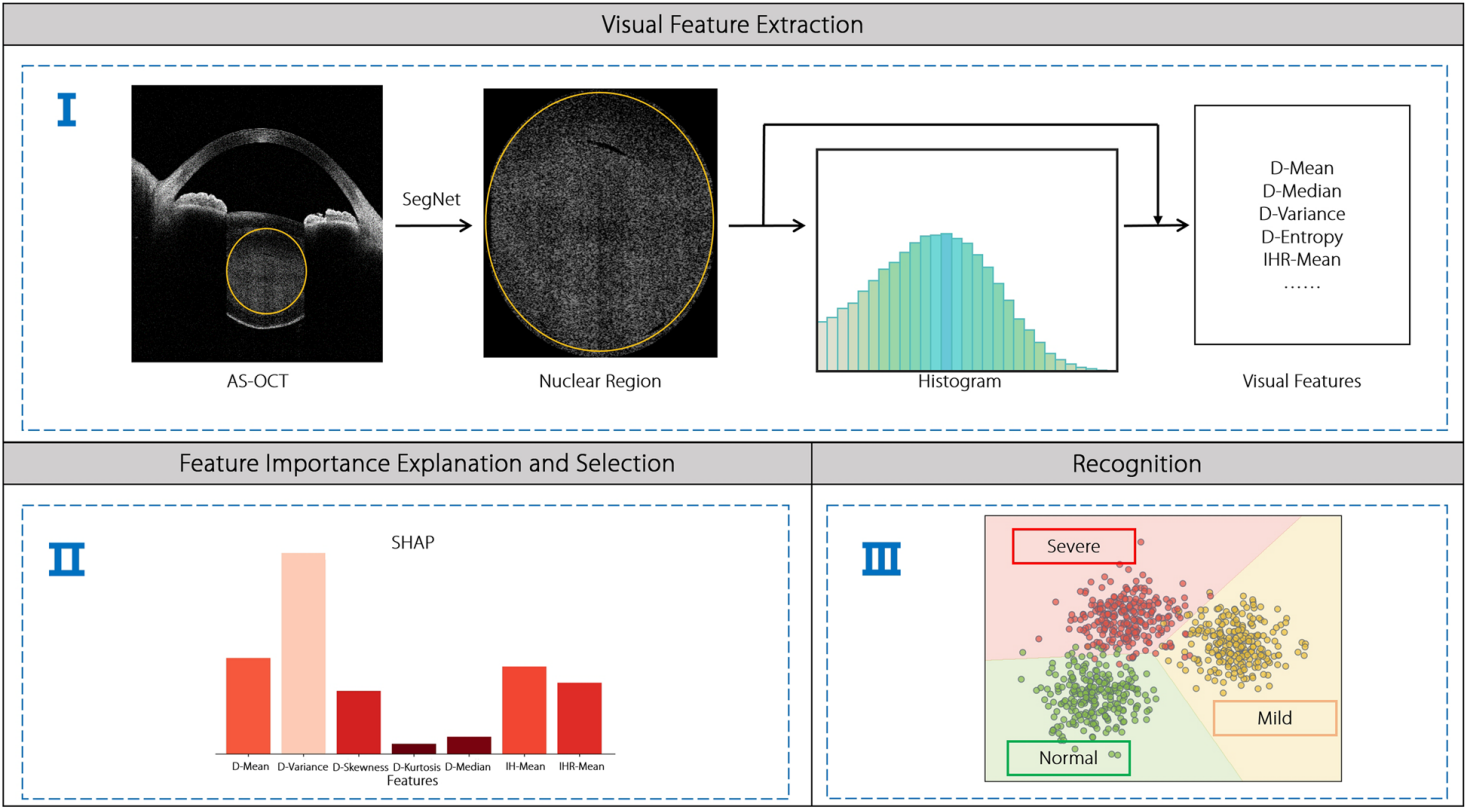
Flowchart of the proposed explainable machine learning framework. Given an AS-OCT image, a deep segmentation network was first applied to segment the nucleus region from AS-OCT images automatically. Secondly, 23 histogram-based statistical features from the AS-OCT-based histogram and four clinical intensity-based statistical features from the original AS-OCT image were extracted. Subsequently, the relative importance of the features and select an informative feature set based on SHAP and PCC was analyzed. Finally, the EMRR to recognize the cataracts severity level was proposed

Motivated by the above analysis, an explainable machine learning framework for automated cataracts recognition was developed, with the aim of fully leveraging the potential of visual features aggregated from both AS-OCT-based histograms and original AS-OCT images. The proposed framework comprises three main stages: visual feature extraction, feature importance explanation and selection, and recognition (Fig. 2). In the visual feature extraction stage, the intensity distributions in the nucleus region are first analyzed, and the intensity range is selected for AS-OCT-based histogram construction. Subsequently, 23 histogram-based and four clinical intensity-based statistical features are extracted from AS-OCT-based histograms and original AS-OCT images through the intensity histogram and intensity-based statistical methods, respectively; thus, the total number of extracted visual features is 27. Following the feature importance explanation and selection step, the SHapley Additive exPlanations (SHAP) [15] and the Pearson correlation coefficient (PCC) method are applied to estimate the relative importance of the visual features and remove redundant visual features. Finally, an ensemble multi-class ridge regression (EMRR) method is proposed to recognize cataracts severity levels automatically based on the selected visual features. Extensive experiments on a clinical AS-OCT-NC dataset were conducted. The results showed that the framework achieved competitive cataracts recognition performance through comparisons with state-of-the-art (SOTA) DNNs with good explanations and less complexity, meeting the requirements of clinical diagnosis.

The main contributions of this study are summarized as follows:

- This study is the first to analyze the visual difference between original AS-OCT images and AS-OCT-based histograms systematically, suggesting that visual NC severity level differences are more apparent under AS-OCT-based histograms. In addition, extracting visual features from them is suggested to be beneficial to cataracts recognition performance and improves the explanation.
- An explainable machine learning framework for cataracts recognition that consists of visual feature extraction, feature importance explanation and selection, and recognition is developed. Specifically, the SHAP and PCC methods are applied to analyze the importance of visual features in selecting useful visual features. The EMRR method is designed to boost the cataracts recognition performance further.
- The experimental results show that the proposed method achieves competitive cataracts recognition performance and is more explainable than advanced DNNs.

### Related works

In the past several decades, researchers have developed a number of computer-aided medical diagnosis techniques to assist ophthalmologists in grading cataracts severity levels through various ophthalmic imaging modalities [2, 16]. Huang et al. [17] developed an automatic NC grading system based on slit-lamp images and achieved an average prediction error of 0.36. Xu et al. [18] proposed a group sparse regression method for NC grading. Caixinha et al. [19] utilized a support vector machine (SVM) to classify hard cataracts based on ultrasound images. Cao et al. [20] proposed an improved Haar wavelet transform method for automatic cataracts screening in fundus images. With the advent of deep learning techniques, Gao et al. [21] proposed a hybrid DNN for NC grading based on a combination of the convolutional neural network (CNN) and recurrent neural network (RNN). Xu et al. [22] used the Faster RCNN network framework for nucleus region location and NC grading based on slit-lamp images.

In recent years, researchers have achieved significant progress in automated cataracts recognition with AS-OCT images [23–30]. Zhang et al. [23] extracted global and local clinical features from the nucleus region and then applied ensemble multi-class logistic regression (LR) to classify the severity levels of NC. The mixed pyramid attention network [24] and a region-based integration-and-recalibration network (RIRNet) [25] were developed to boost NC recognition performance. Wu et al. [28] developed the efficient DMINet to recognize NC. Xiao et al. [27] proposed a local-global spatial attention network for CC classification. Subsequently, the multi-style spatial attention network [26] was devised to improve the CC classification performance. Unfortunately, most existing methods are dedicated to constructing sophisticated DNN architectures, which easily overlook the explanation requirements in clinical diagnostic practice. Moreover, the visual features extracted from AS-OCT-based histograms provide good explanations that have not been explored to improve cataracts recognition performance.

## Methods

In this study, an explainable machine learning framework for cataracts recognition was developed to exploit the visual features extracted from AS-OCT-based histograms and original AS-OCT images fully, as illustrated in Fig. 2. The framework comprises three steps: visual feature extraction, feature importance explanation, and recognition, which are described in the following subsections.

### Visual feature extraction

As shown in Fig. 2, in the visual feature extraction step, a deep segmentation network to segment the nucleus region based on AS-OCT images automatically is applied. As shown in Fig. 1, visual discrepancies in NC severity levels on AS-OCT-based histograms are more evident than those in the original AS-OCT images. In particular, this study extracts 23 histogram-based statistical features from AS-OCT-based histograms and four clinical intensity-based features from original AS-OCT images. Table 1 lists the definitions of the notations used in this study.

**Table 1:** The definitions of the notations

Notation	Meaning
*N*	Total number of histogram intervals
*i*	Interval index, increasing from left to right, starting with 1
$X_i$	Ratio of intensity in the *i*-th interval among the total intensity
$L_i$	Left boundary value of the *i*-th interval
$\mu$	Discretization mean
*m*	Discretization median
$\sigma$	Discretization of the standard deviation

#### Histogram-based statistical features

The 23 extracted histogram-based statistical features are described as follows:

1. Discretized intensity mean (D-Mean): The mean value of the AS-OCT-based histogram, which can be used as a clinical reference to assess the NC severity level.
  1 $$\begin{aligned} \mu =\sum _{i=1}^{N} i X_{i} \end{aligned}$$
2. Discretized intensity variance (D-Variance): The variance value of the AS-OCT-based histogram, which measures how far the discretized intensity is spread out from the average.
  2 $$\begin{aligned} \sum _{i=1}^{N} (i-\mu )^{2} X_{i} \end{aligned}$$
3. Discretized intensity skewness (D-Skewness): The skewness value of the AS-OCT-based histogram, which measures the asymmetry of the discretized intensity.
  3 $$\begin{aligned} \frac{\sum _{i=1}^{N}(i-\mu )^{3} X_{i}}{(\sum _{i=1}^{N}(i-\mu )^{2} X_{i})^{\frac{3}{2}}} \end{aligned}$$
4. Discretized intensity kurtosis (D-Kurtosis): The kurtosis value of the AS-OCT-based histogram, which measures the peakedness of the discretized intensity.
  4 $$\begin{aligned} \frac{\sum _{i=1}^{N}(i-\mu )^{4} X_{i}}{(\sum _{i=1}^{N}(i-\mu )^{2} X_{i})^{2}}-3 \end{aligned}$$
5. Median discretized intensity (D-Median): The median value of the AS-OCT-based histogram, which can be used as a clinical reference to assess the NC severity level.
6. Intensity histogram mean absolute deviation (IH-Mean): A variant of D-Mean, which also measures how far the discretized intensity is spread out from the average.
  5 $$\begin{aligned} \sum _{i=1}^{N}\left| i-\mu \right| X_{i} \end{aligned}$$
7. Intensity histogram robust mean absolute deviation (IHR-Mean): A variant of the IH-Mean, which only uses the discretized intensity in the range 10% to 90%.
  6 $$\begin{aligned} \sum _{i=1}^{N,10\%-90\%}\left| i-\mu \right| X_{i} \end{aligned}$$
8. Intensity histogram median absolute deviation (IH-Median): A variant of D-Median, which can also be used as a clinical reference to assess the NC severity level.
  7 $$\begin{aligned} \sum _{i=1}^{N}\left| i-m\right| X_{i} \end{aligned}$$
9. Intensity histogram coefficient of variation (IHC-Variation): The coefficient of variation of the AS-OCT-based histogram, which measures the discretized intensity dispersion.
  8 $$\begin{aligned} \frac{\sigma }{\mu } \end{aligned}$$
10. Discretized intensity entropy (D-Entropy): The entropy value of the AS-OCT-based histogram, which can also be used as a clinical reference to assess the NC severity level.
  9 $$\begin{aligned} -\sum _{i=1}^{N}X_{i} \log _{2}{X_{i}} \end{aligned}$$
11. Discretized intensity uniformity (D-Uniformity): The uniformity value of the AS-OCT-based histogram, which measures the uniformity of the discretized intensity.
  10  $$\begin{aligned} \sum _{i=1}^{N} X_{i}^{2} \end{aligned}$$
12. $10^{th}$ discretized intensity percentile (D-Ten): The histogram interval index where the discretized intensity at $10\%$ lies.
13. $90^{th}$ discretized intensity percentile (D-Ninety): The histogram interval index where the discretized intensity at $90\%$ lies.
14. Intensity histogram mode (IH-Mode): The largest discretized intensity value of the AS-OCT-based histogram.
15. Minimum discretized intensity (D-Minimum): The left boundary of the smallest discretized intensity in the AS-OCT-based histogram.
  11 $$\begin{aligned} L_{min}=min(L_{i}) \end{aligned}$$
16. Maximum discretized intensity (D-Maximum): The left boundary of the largest discretized intensity in the AS-OCT-based histogram.
  12 $$\begin{aligned} L_{max}=max(L_{i}) \end{aligned}$$
17. Discretized intensity interquartile range (D-Interquartile): The interquartile range of the AS-OCT-based histogram, which is the difference between the discretized intensity interquartiles.
  13 $$\begin{aligned} L_{75^{th}}-L_{25^{th}} \end{aligned}$$
  where $L_{25^{th}}$ and $L_{75^{th}}$ are the left boundaries of the intervals where the lower-quartile and upper-quartile discretized intensities lie, respectively.
18.  Discretized intensity range (D-Range): The range of the AS-OCT-based histogram, which is the difference between the maximum and minimum discretized intensity.
  14 $$\begin{aligned} L_{100^{th}}-L_{1^{th}} \end{aligned}$$
  where $L_{1^{th}}$ and $L_{100^{th}}$ are the left boundaries of the smallest and largest intervals, respectively.
19.  Intensity histogram quartile coefficient of dispersion (IHC-Dispersion): The quartile coefficient of dispersion of the AS-OCT-based histogram, which focuses on the histogram dispersion within the upper and lower quartiles.
  15 $$\begin{aligned} \frac{L_{75^{th}}-L_{25^{th}}}{L_{75^{th}}+L_{25^{th}}} \end{aligned}$$
20. Maximum histogram gradient (G-Maximum): The maximum value of the gradient of the AS-OCT-based histogram.
21. Maximum histogram gradient intensity (GI-Maximum): The interval index where G-Maximum lies.
22. Minimum histogram gradient (G-Minimum): The minimum value of the gradient of the AS-OCT-based histogram.
23. Minimum histogram gradient intensity (GI-Minimum): The interval index where G-Minimum lies.

Moreover, two significant problems occur in extracting statistical features from AS-OCT-based histograms: intensity range selection and intensity interval selection, which are determined through a series of experiments.

#### Intensity-based statistical features

In addition to histogram-based statistical features, this study also extracted four clinical intensity-based statistical features from the original AS-OCT images, namely the intensity mean, intensity median, intensity standard deviation, and intensity maximum, based on clinical research and published work [10, 11].

1. Intensity mean (I-Mean): The average value of the pixels in the nuclear area, which is an essential reference indicator for determining the NC severity in clinical diagnosis.
2. Intensity median (I-Median): The median value of the pixels in the AS-OCT image of the nuclear area, which is also an essential reference indicator.
3. Intensity standard deviation (I-STD): The standard deviation is the arithmetic square root of the variance and is used to measure the dispersion (uniformity) of the pixels in AS-OCT images.
4. Intensity maximum (I-Maximum): The maximum value of the pixels in the AS-OCT image of the nuclear area.

### Feature importance explanation and selection

In terms of clinical interpretability and NC recognition performance requirements, this study adopts a combination of the SHAP and PCC methods to explain the relative significance of features and select informative features. SHAP is a widely accepted method for explaining the relative contributions of features to the model, which can also be applied to analyze the importance of features from a global perspective. In contrast, PCC has been used extensively for clinical feature importance analysis from a local perspective. Therefore, the aim is to combine the advantages of both methods through the global and local importance of features for feature selection, thereby boosting interpretability.

**SHAP.** Given the feature set *T* and model $f_S$ trained on the feature subset $S\subseteq T$, SHAP usually assigns an importance value to each feature, which indicates its impact on the model prediction performance. For feature $i\in T$, model $f_{S\bigcup \left\{ i \right\} }$ is retrained on feature set $S\bigcup \left\{ i \right\}$, whereas another model $f_{S}$ is retrained on feature set *S* without feature *i*. Then, SHAP calculates the contribution of feature *i* by computing the difference between the predictions of $f_{S\bigcup \left\{ i \right\} }(y_{S\bigcup \left\{ i \right\} })$ and $f_{S}(y_S)$, where $y_S$ represents the input data in feature set *S*. It searches all subsets *S* without feature *i* to obtain the overall SHAP value, weighted by the feature number in *S*. Thus, a larger SHAP value indicates a greater contribution. The following formula describes the calculation process.

16 $$\begin{aligned} \phi _{i}=\sum _{S\subseteq T\backslash \left\{ i\right\} } \frac{|S|!(|T|-|S|-1)!}{|T|!} \left[ f_{S\bigcup \left\{ i \right\} }(y_{S\bigcup \left\{ i \right\} } )-f_S(y_S) \right] \end{aligned}$$

where $\phi _{i}$ is the SHAP value of the *i*-th feature.

**PCC.** The PCC method can explain the correlation between the two features from a local perspective by calculating the covariance and standard deviation of the two features in the sample. Based on the AS-OCT-NC dataset, PCCs are calculated using the following formula:

17 $$\begin{aligned} PCC_{x,y}=\frac{N\sum _{i}^{N} x_{i}y_{i}-\sum _{i}^{N} x_{i}\sum _{i}^{N} y_{i}}{\sqrt{N\sum _{i}^{N} x_{i}^{2}-(\sum _{i}^{N} x_{i})^{2}} \sqrt{N\sum _{i}^{N} y_{i}^{2}-(\sum _{i}^{N} y_{i})^{2}} } \end{aligned}$$

where *x* and *y* denote the two features, and *N* and *i* are the total samples and sample index, respectively.

### Recognition

**Fig. 3:**
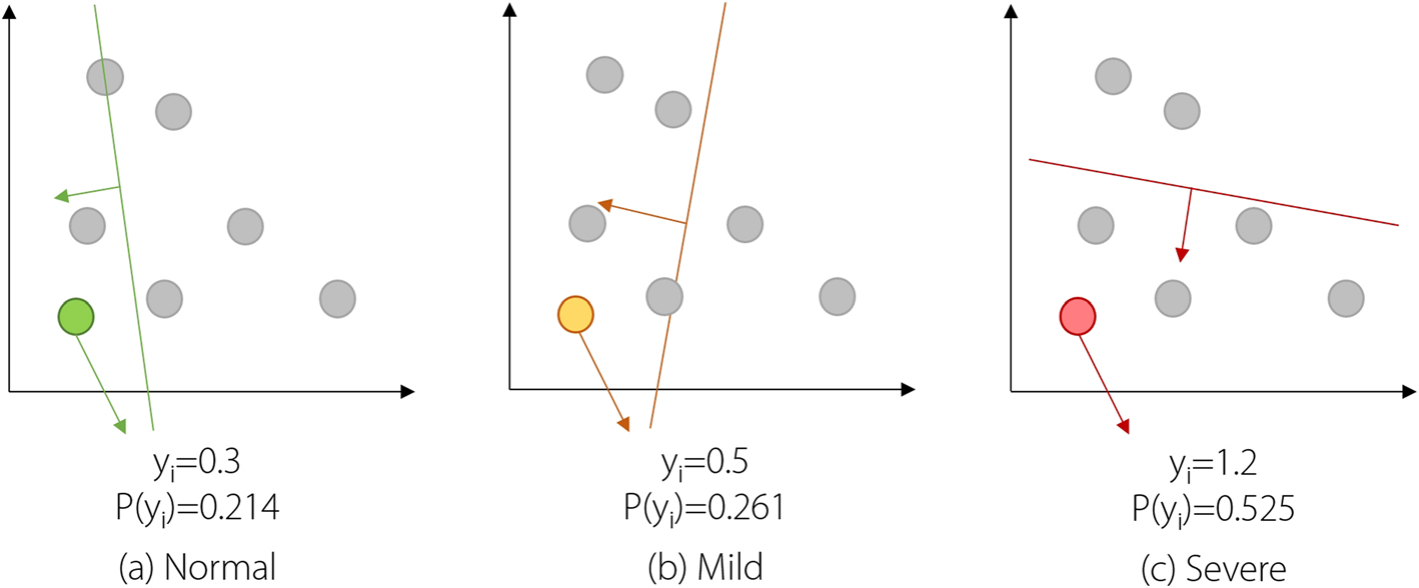
RR models for different NC severity levels: **a** normal, **b** mild, and **c** severe. The EMRR first calculates the $P(y_{i})$ for each level based on the corresponding model output $y_{i}$. Subsequently, it selects the level with the largest $P(y_{i})=0.525$ as the final output, which is severe

This study proposes the EMRR method for automatic NC recognition based on the selected visual features. EMRR comprises of multiple ridge regression (RR) models that aim to leverage the high interpretability and fast computation time of RR. In particular, each RR model predicts the probability for each class, as shown in Fig. 3. The probability for each class is calculated as follows:

18 $$\begin{aligned} P(y=i|\textbf{x})=\frac{e^{y_{i}(\textbf{x})}}{\sum _{j=1}^{C} e^{y_{j}(\textbf{x})}} \end{aligned}$$

19 $$\begin{aligned} y_{i}(\textbf{x})= \varvec{\omega }^{\textbf{T}} \textbf{x}+\omega _{0}=\omega _{1}x_{1}+\omega _{2}x_{2}+\omega _{3}x_{3}+\ldots +\omega _{M}x_{M}+\omega _{0} \end{aligned}$$

where $P(y=i|\textbf{x})$, $\textbf{x}$, $\mathbf {\omega }$, $y_{i}(\textbf{x})$, and *M* denote the probability, visual feature vector, learned coefficients, output value, and total number of features, respectively. Assuming that *C* is the total number of classes, EMRR selects class *k* as the final decision based on the one-vs-all strategy:

20 $$\begin{aligned} P(y=k|\textbf{x})>P(y=j|\textbf{x}), \forall i,k\in \left\{ 1,\ldots ,C \right\} \ and\ i\ne k \end{aligned}$$

During the training process, the training samples $\textbf{X}$, coefficients $\hat{\varvec{\omega }}$, and ground truth $\textbf{Y}$ were compresses into the following formulas:

21 $$\begin{aligned} X= \left[ \begin{array}{ccccc} x_{1,1}^{T} & x_{1,2}^{T} & \cdots & x_{1,M}^{T} & 1\\ x_{2,1}^{T} & x_{3,2}^{T} & \cdots & x_{2,M}^{T} & 1\\ \vdots & \vdots & \ddots & \vdots & \vdots \\ x_{N,1}^{T} & x_{N,2}^{T} & \cdots & x_{N,M}^{T} & 1 \end{array}\right] = \left[ \begin{array}{cc} \textbf{x}_{\varvec{1}}^{\textbf{T}} & 1\\ \textbf{x}_{\varvec{2}}^{\textbf{T}} & 1\\ \vdots & \vdots \\ \textbf{x}_{\varvec{N}}^{\textbf{T}} & 1 \end{array}\right] \end{aligned}$$

22 $$\begin{aligned} \hat{\varvec{\omega }}=(\omega _{1}, \omega _{2}, \omega _{3}, \ldots , \omega _{M}, \omega _{0})^{T}= \left[ \begin{array}{c} \mathbf {\omega }\\ \mathbf {\omega _{0}} \end{array}\right] \end{aligned}$$

23 $$\begin{aligned} Y= \left[ \begin{array}{c} y_{1}\\ y_{2}\\ \vdots \\ y_{N}\\ \end{array}\right] \end{aligned}$$

where *N* denotes the total number of training samples. The ground truth for each RR model is formulated as follows: 1 for the correct class and 0 for the incorrect class. The ordinary least squares method with an $L_{2}$ regularization is then used as the objective function, summarized as follows:

24 $$\begin{aligned} \min _{\hat{\omega }} \left\| \textbf{X}\hat{\varvec{\omega }}-Y \right\| _{2}^{2}+\lambda \left\| \hat{\varvec{\omega }} \right\| _{2}^{2} \end{aligned}$$

25 $$\begin{aligned} \hat{\varvec{\omega }}=(X^{T}X+\lambda I)^{-1}X^{T}Y \end{aligned}$$

where $\lambda$ is the penalty coefficient that controls the scalar of coefficients and *I* is the identity matrix. The above objective function is used to train each RR model independently and combine them in the final prediction stage.

### Dataset and experimental setup

#### AS-OCT-NC dataset

In this study, the clinical AS-OCT-NC dataset, collected by CASIA2 (TOMEY Inc, Japan), is used to validate the effectiveness of the proposed framework. The dataset includes 483 participants (336 right and 350 left eyes) with 12,824 AS-OCT images. The average age of the participants was $61.30\pm 18.65$ years. The dataset collection was conducted in accordance with the tenets of the Declaration of Helsinki. Owing to the retrospective nature and fully anonymized usage of the dataset, the medical ethics committee granted an exemption from informing the patients. For the machine learning methods, the AS-OCT-NC dataset was split into training (9,032) and test (3,792) sets based on the participant-level. All machine learning methods were trained until convergence on the training set and then tested on the test set. For the DNNs, 20% of the training set was used as the validation set to select the best-trained model. The dataset distributions for the DNNs are listed in Table 2.

**Table 2:** Distribution of images by different NC level images for deep learning methods

Set	Normal	Mild	Severe	Total
Training	760	2,233	4,233	7,226
Validation	190	558	1,058	1,806
Test	287	1,102	2,403	3,792
Total	1,237	3,893	7,694	12,824

#### Evaluation metrics

Following previous studies [8, 9], five commonly accepted evaluation measures to assess the performance of the methods were used: accuracy (ACC), precision (PRE), sensitivity (SEN), specificity (SPE), and F1 score (F1). These evaluation metrics are formulated as follows:

26 $$\begin{aligned} ACC=\frac{TP+TN}{TP+FP+TN+FN} \end{aligned}$$

27 $$\begin{aligned} PRE=\frac{TP}{TP+FP} \end{aligned}$$

28 $$\begin{aligned} SEN=\frac{TP}{TP+FN} \end{aligned}$$

29 $$\begin{aligned} SPE=\frac{TN}{TN+FP} \end{aligned}$$

30 $$\begin{aligned} F1=\frac{2\times PRE\times SEN}{PRE+SEN} \end{aligned}$$

where TP, TN, FP, and FN denote the numbers of true positives, true negatives, false positives, and false negatives, respectively.

#### Experimental settings

The OpenCV, scikit-learn, and SHAP packages were used to implement the framework. The machine learning methods were trained until convergence. The comparable DNNs were implemented using the PyTorch platform and trained from scratch using stochastic gradient descent (SGD) optimized with the default settings. The learning rate, training epochs, batch size, and image size were set to 0.001, 100, 32, and 224, respectively. Additionally, the initial learning rates were set to 0.1, 0.01, and 0.001, and the batch sizes were set to 32 and 64 for hyperparameter selection. The learning rate was reduced by a factor of 10 every 50 epochs. All the experiments were performed on a server with an Intel(R) Xeon(R) Gold 6138 CPU and an NVIDIA TITAN V GPU (12 GB). For the DNNs, the channel means and standard deviations were set to 0.5 following the literature [25] for both the training and test sets. The original AS-OCT image was resized to 224 $\times$ 224. In addition, the rotation ($-10^{\circ }$, $10^{\circ }$) and flip (horizontal and vertical directions) methods were utilized to augment the training set.

#### Factor effect settings of the proposed framework

In this study, several factors affecting the automated cataracts recognition performance of the proposed framework were investigated.

- **Effects of intensity range selection on histogram-based statistical features**: The intensity values of the nucleus region vary depending on their characteristics, as shown in Fig. 1. The zero-intensity values occupy a significant proportion of the total values, while those over 150 only occupy a small proportion. The intensity range selection for constructing AS-OCT-based histograms has a significant impact on histogram-based statistical features. Moreover, the influence of intensity range selection was investigated by setting two different intensity ranges: 0–255 and 5–150.
- **Effects of intensity interval selection on histogram-based statistical features**: In AS-OCT-based histograms, the intensity interval selection also affects the histogram-based statistical feature distributions of different NC severity levels. For example, a small intensity interval leads to finer information distribution. Two intensity intervals were set for comparison: 5 and 10.
- **Effects of different feature selection methods**: Feature selection aims to select important features while removing redundant features, significantly improving the cataracts recognition performance of the model. Linear discriminant analysis (LDA), principal component analysis (PCA), recursive feature elimination (RFE), and recursive feature augmentation (RFA) are the commonly used feature selection methods. These methods were applied to test the effectiveness of the proposed feature explanation and selection method.

#### Baselines

To prove the effectiveness of the proposed method, classical machine learning methods and advanced DNNs were adopted for comparison.

**Machine learning methods**: naive Bayes (NB), RR, LR, SVM with linear kernel (SVM-Linear), SVM with radial basis function kernel (SVM-RBF), K-nearest neighbors (KNN), decision tree (DT), random forest (RF), AdaBoost, and gradient boosting (GB).

**DNNs specifically designed for cataracts recognition**: GraNet [12], AFSNet [9], GCANet [13], RIRNet [25], and RCRNet [8].

**Commonly-used yet advanced DNNs**: AlexNet [31], VGGNet [32], ResNet [33], ResNeXt [34], SENet [35], SRMNet [36], EfficientNet [37], MobileNetV2 [38], ShuffleNetV2 [39], ViT [40], and MLPMixer [41].

## Results and Discussion

### Feature importance analysis and explanation

SHAP [15] and PCC methods were applied to analyze the relative significance of 27 visual features extracted from AS-OCT-based histograms and original AS-OCT images to select significant visual features and remove redundant visual features. First, the EMRR was trained on the training set. Subsequently, the SHAP value of each visual feature was calculated based on the EMRR to analyze their contributions from a global perspective. The PCC values among the visual features and NC severity levels were also obtained based on the training set, assisting in selecting informative features from a local perspective.

Table 3 lists the SHAP values, PCC values, and recognition accuracies of each feature. The PCC values mainly measure the correlations between the severity levels of the NC and visual features. Thus, the focus was on analyzing SHAP values of the extracted visual features. I-STD had the highest SHAP value. D-Variance and IH-Mean achieved the highest ACC. Notably, IHC-Variation had the highest PCC value of 0.832, but exhibited relatively poor cataracts recognition performance. The small SHAP value of IHC-Variation also indicates that the PCC values may be affected by other features. In contrast, D-Minimum had zero SHAP and PCC values and the lowest recognition ACC, indicating that it had a negligible effect on the NC recognition performance. D-Uniformity had the second lowest SHAP value, possibly because the pixel values were concentrated at lower intervals, causing steady uniformity. This phenomenon may also have led to the failure of D-Minimum.

**Table 3:** Overall absolute SHAP values, PCC values, and corresponding recognition accuracies of each feature

Index	Name	SHAP	PCC	ACC (%)	Index	Name	SHAP	PCC	ACC (%)
1	D-Mean	0.639	0.695	77.98	15	D-Minimum	0.000	0.000	63.37
2	D-Variance	1.335	0.614	**78.53**	16	D-Maximum	0.018	0.436	69.65
3	D-Skewness	0.420	0.802	71.73	17	D-Interquartile	0.037	0.648	74.87
4	D-Kurtosis	0.068	0.697	69.49	18	D-Range	0.018	0.436	69.65
5	D-Median	0.115	0.701	75.40	19	IHC-Dispersion	0.030	0.714	69.49
6	IH-Mean	0.582	0.670	**78.53**	20	G-Maximum	0.154	0.588	70.19
7	IHR-Mean	0.474	0.663	77.56	21	GI-Maximum	0.160	0.649	70.78
8	IH-Median	0.563	0.723	77.40	22	G-Minimum	0.164	0.660	69.73
9	IHC-Variation	0.059	**0.832**	70.75	23	GI-Minimum	0.164	0.818	72.86
10	D-Entropy	0.383	0.772	75.08	24	I-Mean	1.391	0.692	78.11
11	D-Uniformity	0.010	0.795	69.17	25	I-Median	0.129	0.704	76.77
12	D-Ten	0.226	0.613	70.94	26	I-STD	**1.675**	0.647	78.30
13	D-Ninety	0.314	0.682	77.56	27	I-Maximum	0.023	0.355	65.88
14	IH-Mode	0.365	0.735	77.40					

**Fig. 4:**
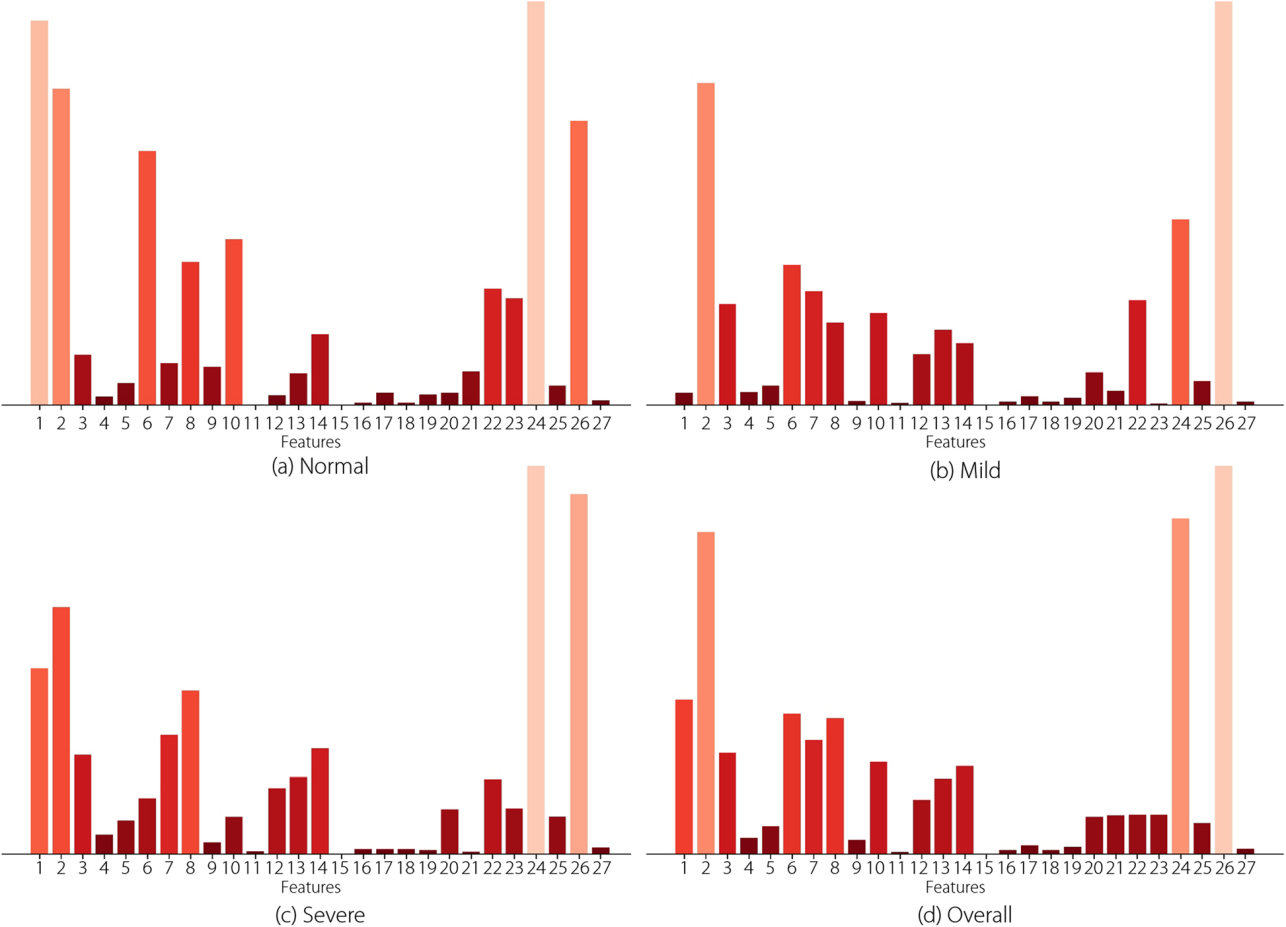
SHAP values between the 27 visual features and NC severity levels: **a** normal, **b** mild, **c** severe, and **d** overall. The visual features contributed differently to recognizing different NC severity levels, and the height of each denotes their importance

**Fig. 5:**
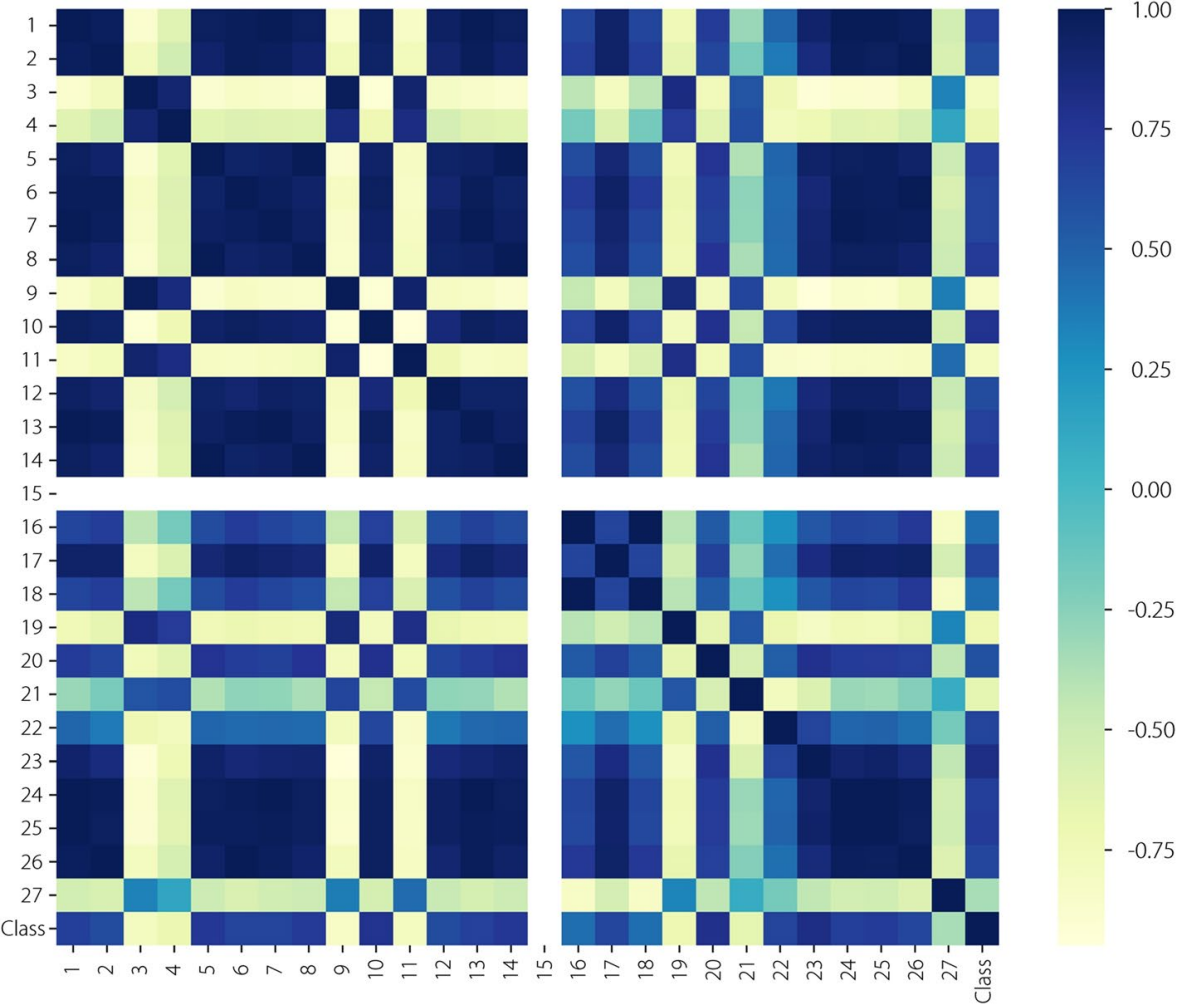
PCC matrix between features. The coefficients ranged from -1 to 1, indicating the degree of correlation. Values close to -1 and 1 indicate a high correlation, whereas values close to 0 indicate a low correlation

To investigate the relative roles of each feature in the NC recognition results further, an in-depth analysis of the relationships between the SHAP values of the 27 visual features and NC severity levels was conducted, as shown in Fig. 4. It can be observed that D-Mean and I-STD performed better in normal and severe NC recognition, whereas IH-Mean and D-Entropy performed better in normal and mild NC recognition. Figure 5 lists the PCC values among all visual features. The PCC values between D-Minimum and the other features were zero because D-Minimum is zero by nature. G-Maximum, G-Minimum, and D-Median were highly correlated with GI-Maximum, GI-Minimum, and I-Median, indicating that they may play similar roles in cataracts recognition performance.

According to the above analysis, the D-Minimum, D-Uniformity, G-Maximum, G-Minimum, and D-Median were eliminated because they had negligible effects on the NC recognition performance. Therefore, 22 visual features were selected for automatic NC recognition.

Table 4 presents the NC recognition result comparisons based on the original and selected visual features. It can be observed that the five machine learning methods achieved worse NC recognition results using the original visual features than those using the selected visual features, indicating that the selected visual features were more significant than the deleted visual features. The EMRR performed better than other machine learning methods based on both the original and selected visual features, proving the superiority and generalizability of the proposed approach.

**Table 4:** Comparisons of cataracts recognition results between original and selected feature sets using five machine learning methods

Method	Original					Selected				
	ACC (%)	SEN (%)	PRE (%)	SPE (%)	F1 (%)	ACC (%)	SEN (%)	PRE (%)	SPE (%)	F1 (%)
LR	90.61	90.28	93.42	92.31	91.46	91.56	91.57	93.60	93.39	92.42
SVM-Linear	90.72	90.52	93.31	92.54	91.64	90.82	89.73	94.64	91.99	91.42
RF	89.21	90.02	91.27	91.92	90.54	90.14	90.51	92.20	92.48	91.23
KNN	86.68	87.17	89.24	89.94	88.03	88.00	88.85	90.19	91.18	89.45
EMRR	91.30	91.00	94.00	92.82	92.14	**92.80**	**93.10**	**94.39**	**94.46**	**93.65**

### Comparisons with advanced methods

Table 5 presents the NC recognition results of the SOTA machine learning methods, DNNs, and the proposed EMRR. The EMRR achieved the best results for the four evaluation measures: 92.80% ACC, 93.10% SEN, 94.46% SPE, and 93.65% F1. In particular, the EMRR performed better than RR, demonstrating its superiority in exploiting the potential of a set of RR classifiers based on the voting strategy. Compared with GraNet, AFSNet, GCANet, RIRNet, and RCRNet, which are specifically designed for NC recognition based on AS-OCT images, the proposed method achieved absolute 1.4%, 3.11%, and 1.99% gains in ACC, SEN, and F1, respectively. Remarkably, the EMRR significantly outperformed the recent ViT and MLPMixer by over 8.43%, 2.84%, and 6.36% in terms of SEN, PRE, and F1, respectively. Moreover, the machine learning methods achieved competitive performance through comparisons with DNNs, with fewer parameters and lower computational costs.

**Table 5:** NC classification results of the EMMR, competitive machine learning methods, and SOTA DNNs

Method	ACC (%)	SEN (%)	PRE (%)	SPE (%)	F1 (%)
GraNet	90.03	89.98	92.08	92.67	90.98
AFSNet	91.67	90.56	93.76	93.60	92.04
GCANet	89.00	85.64	84.61	94.22	84.92
RIRNet	89.43	85.57	84.48	94.11	84.96
RCRNet	91.03	81.94	92.36	94.22	85.60
AlexNet	91.06	86.92	92.74	93.45	89.53
VGGNet-13	91.40	92.73	92.59	94.27	92.65
ResNet-18	89.32	86.14	91.55	92.15	88.59
ResNeXt	90.93	90.95	92.66	93.09	91.69
SENet	90.24	90.44	91.32	92.70	90.77
SRMNet	91.30	91.39	93.00	93.35	92.09
EfficientNet	91.54	91.21	93.60	93.25	92.19
MobileNetV2	91.27	87.35	92.70	94.05	89.70
ShuffleNetV2	91.01	92.06	92.23	93.79	92.14
ViT	91.59	82.20	91.47	94.20	85.81
MLPMixer	91.59	83.69	89.94	94.45	86.30
NB	85.36	91.15	88.27	92.70	88.46
RR	91.32	91.45	93.32	93.26	92.24
LR	91.56	91.57	93.60	93.39	92.42
SVM-Linear	90.82	89.73	**94.64**	91.99	91.42
SVM-RBF	90.11	89.70	89.81	92.64	89.47
KNN	88.00	88.85	90.19	91.18	89.45
DT	86.08	86.96	88.77	89.45	87.66
RF	90.14	90.51	92.20	92.48	91.23
AdaBoost	87.13	86.58	91.21	89.28	88.02
GB	91.32	91.45	93.32	93.26	92.24
EMRR	**92.80**	**93.10**	94.39	**94.46**	**93.65**

Table 6 lists the NC recognition results of five advanced DNNs with different learning rates and batch sizes on the AS-OCT-NC dataset. The performance with a learning rate of 0.001 and batch size of 64 was slightly better, but it doubled the memory required to train the DNNs. Nevertheless, their ACC and F1 were still lower than those of the proposed EMRR.

**Table 6:** NC classification results of DNNs with different hyperparameters

Method	Learning rate (%)	Batch size (%)	ACC (%)	SEN (%)	PRE (%)	SPE (%)	F1 (%)
AFSNet	0.1	32	91.19	90.59	93.93	92.87	92.00
		64	91.56	93.06	92.53	94.53	**92.77**
	0.01	32	90.82	88.75	94.19	92.26	90.95
		64	91.27	92.02	92.86	93.81	92.43
	0.001	32	91.67	90.56	93.76	93.60	92.04
		64	91.30	92.83	91.90	94.44	92.33
ResNeXt	0.1	32	89.00	87.68	93.53	90.52	89.69
		64	91.40	91.53	92.77	93.74	92.12
	0.01	32	89.90	89.73	92.34	92.26	90.90
		64	90.88	90.41	93.57	92.69	91.75
	0.001	32	90.93	90.95	92.66	93.09	91.69
		64	91.32	90.55	93.79	93.13	91.98
SRMNet	0.1	32	90.59	90.56	92.41	93.21	91.45
		64	91.43	90.18	94.41	92.81	91.89
	0.01	32	90.59	90.72	92.07	93.39	91.38
		64	90.45	89.49	88.59	92.81	88.17
	0.001	32	91.30	91.39	93.00	93.35	92.09
		64	**91.88**	91.23	94.51	93.37	92.63
MobileNetV2	0.1	32	91.06	89.63	**95.06**	92.11	91.56
		64	90.64	92.93	90.82	94.43	91.78
	0.01	32	89.43	89.95	91.14	92.40	90.52
		64	89.35	89.71	91.78	91.92	90.63
	0.001	32	91.27	87.35	92.70	94.05	89.70
		64	91.43	**93.97**	91.62	**95.03**	92.64
MLPMixer	0.1	32	88.69	78.47	91.58	91.03	83.42
		64	84.57	59.06	84.24	89.94	56.54
	0.01	32	90.98	86.25	92.83	93.35	89.16
		64	91.54	81.59	92.10	94.05	91.24
	0.001	32	91.59	83.69	89.94	94.45	86.30
		64	91.83	85.68	93.50	94.08	89.00

Table 7 presents the results of the EMRR, LR, and four representative DNNs on the AS-OCT-NC dataset based on five-fold cross-validation. First, the AS-OCT-NC dataset was divided into five disjointed sets. Subsequently, at each time point, four sets were used for training and one set was used for testing. The results of the DNNs are presented in the “Average (standard deviation)” form. In comparison, EMRR performed better and was more robust.

**Table 7:** NC classification results of the EMRR, LR, and DNNs with standard deviation

Method	ACC (%)	SEN (%)	PRE (%)	SPE (%)	F1 (%)
AFSNet	$84.59\pm 2.24$	$86.68\pm 3.21$	$86.96\pm 1.65$	$89.87\pm 2.45$	$86.65\pm 2.32$
EfficientNet	$83.45\pm 3.02$	$83.16\pm 4.35$	$86.14\pm 1.70$	$89.07\pm 2.16$	$84.38\pm 3.22$
ViT	$82.26\pm 2.62$	$81.25\pm 4.63$	$81.47\pm 2.75$	$89.07\pm 1.92$	$81.08\pm 3.42$
MLPMixer	$82.22\pm 2.33$	$80.32\pm 2.94$	$81.20\pm 3.79$	$88.76\pm 1.61$	$80.60\pm 2.95$
LR	$86.42\pm 0.62$	$88.71\pm 0.69$	$88.59\pm 0.58$	$91.12\pm 0.48$	$88.64\pm 0.60$
EMRR	**87.17 ** $\varvec{\pm }$** 0.57**	**90.00 ** $\varvec{\pm }$** 0.37**	**89.02 ** $\varvec{\pm }$** 0.48**	**92.00 ** $\varvec{\pm }$** 0.30**	**89.46 **± **0.42**

**Fig. 6:**
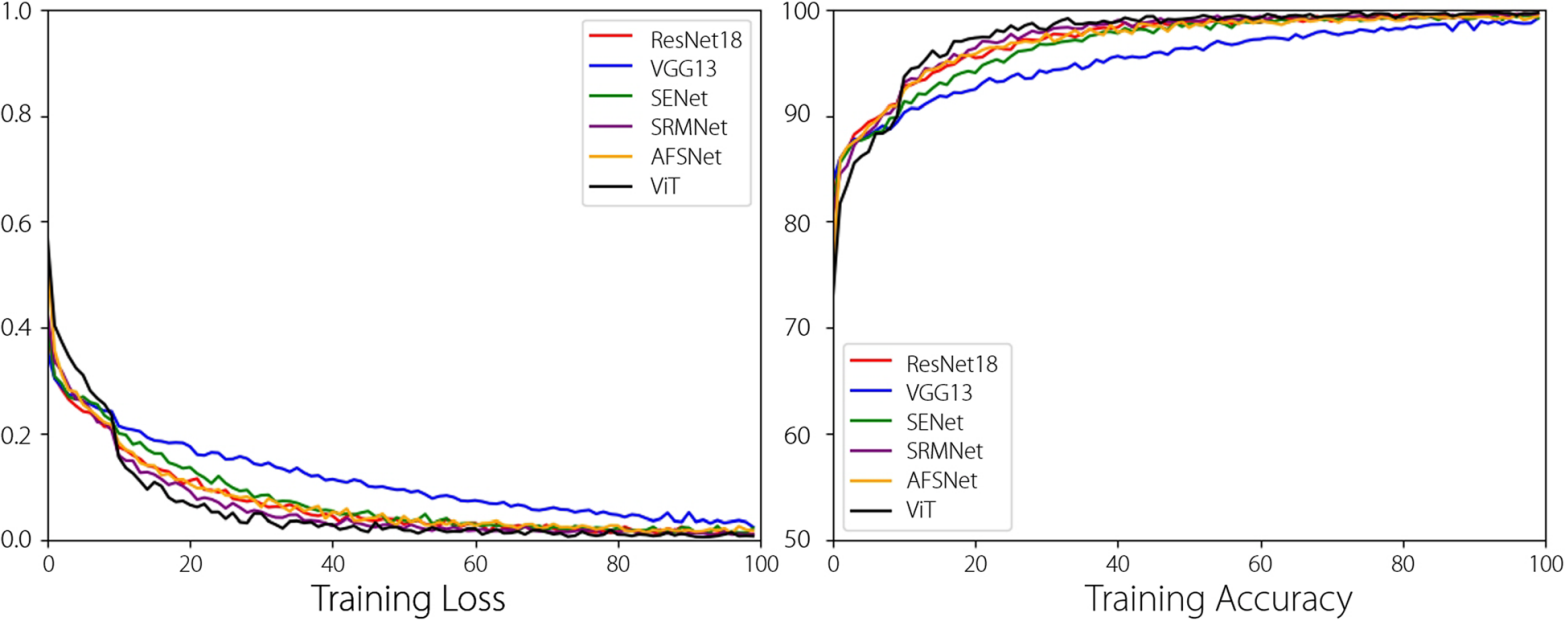
Training loss and ACC of different DNNs with a learning rate of 0.001

Figure 6 presents the training processes of two traditional DNNs, two attention-based DNNs, one cataract-based DNN, and one Transformer-based DNN with a learning rate of 0.001. All networks converged after 80 epochs.

**Fig. 7:**
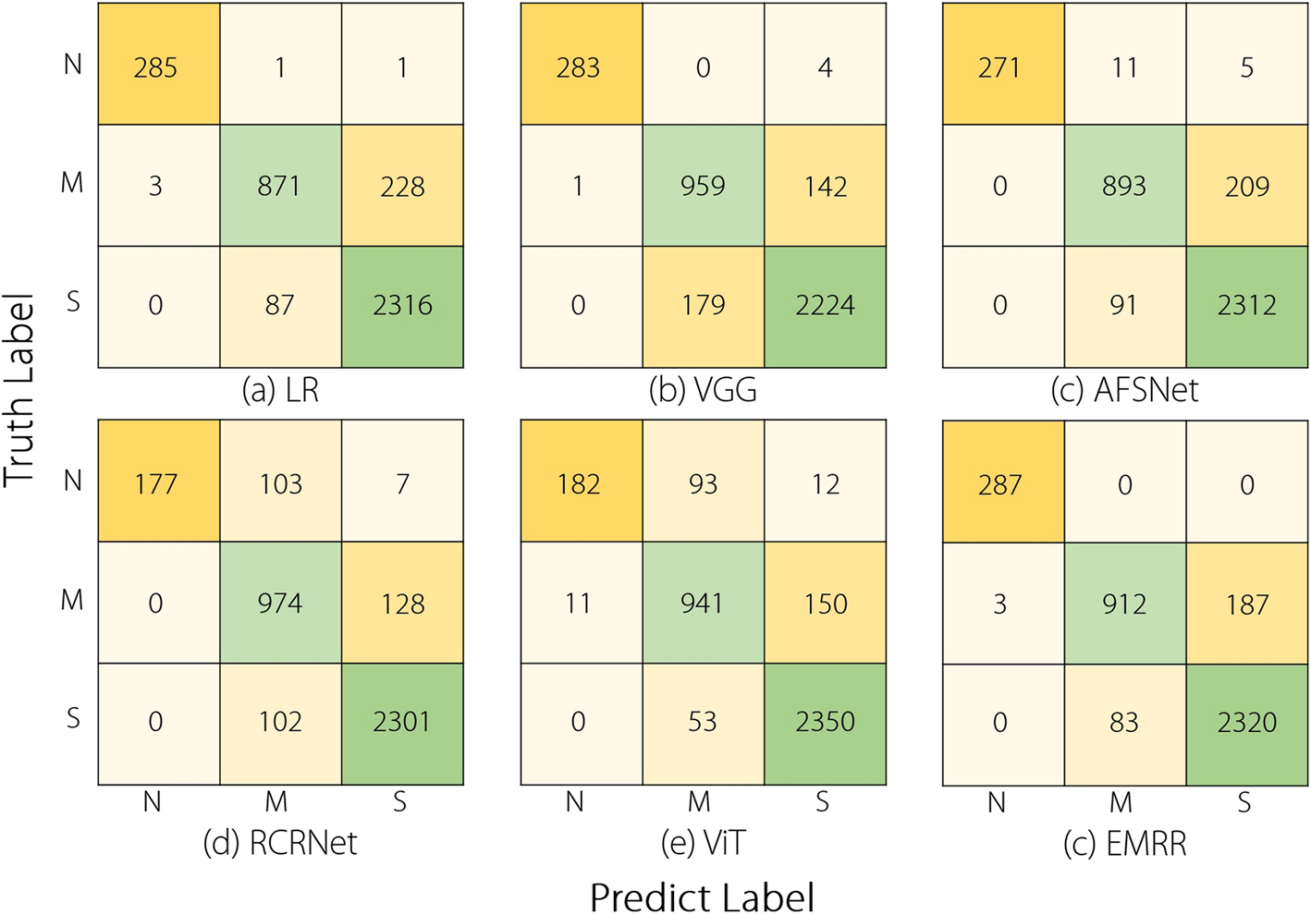
Confusion matrices of the EMRR, LR, and four other DNNs on AS-OCT-NC dataset with a learning rate of 0.001

Figure 7 shows the confusion matrices of the proposed EMRR, LR, and four representative DNNs with a learning rate of 0.001. The proposed EMRR achieved better normal case recognition results than the other five comparable methods, which explains why the proposed method obtained higher SPE values than the others. However, this method performed worse than RCRNet, VGG, and ViT for mild NC recognition, indicating room for improvement.

### Ablation study

#### Effects of intensity range selection on histogram-based statistical features

**Fig. 8:**
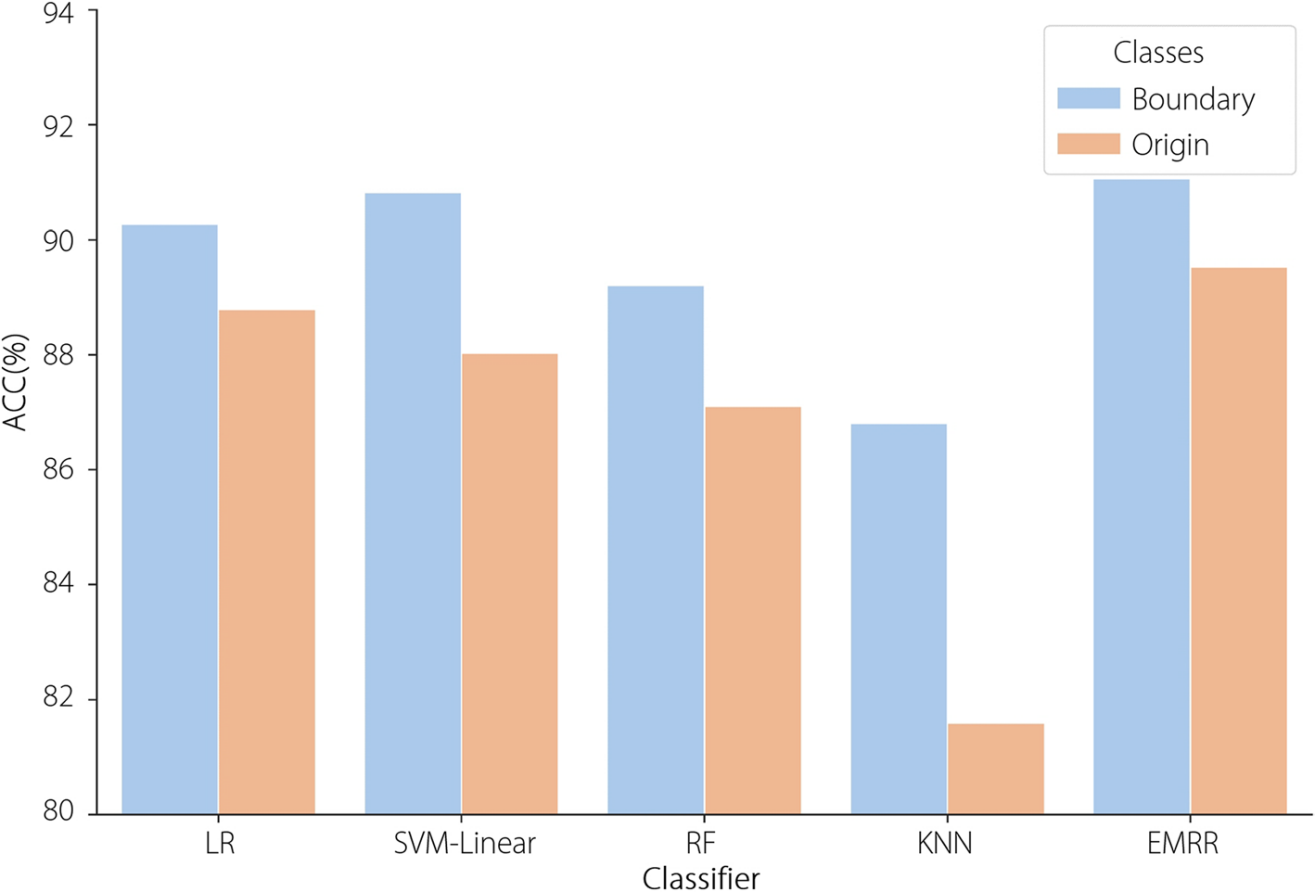
Performance comparison of machine learning methods based on histogram-based statistical features in different intensity ranges

Table 8 and Fig. 8 present cataracts recognition result comparisons of histogram-based statistical features based on two intensity range selection settings: 0–255 (original) and 5–150 (boundary). In this study, five machine learning methods were adopted, including EMRR, for cataracts recognition. The five machine learning methods based on histogram-based statistical features extracted from AS-OCT-based histograms in the intensity range 5–150 significantly outperformed those in the intensity range 0–255. The possible reasons for the results are as follows: (1) AS-OCT-based histograms constructed in the intensity value range of 0–255: the zero intensity values occupy a significant proportion of the total values, while those over 150 only occupy a small proportion, which leads to histogram-based statistical features among different cataracts severity levels being small because of redundant feature information. (2) AS-OCT-based histograms constructed in the intensity value range of 5–150: histogram-based statistical features among different cataracts severity levels are apparent, making it easy for machine learning methods to recognize different cataracts severity levels.

**Table 8:** Performance comparison of machine learning methods based on histogram-based statistical features in different intensity ranges

Method	Original					Boundary				
	ACC (%)	SEN (%)	PRE (%)	SPE (%)	F1 (%)	ACC (%)	SEN (%)	PRE (%)	SPE (%)	F1 (%)
LR	88.79	87.17	90.24	90.76	87.57	90.27	89.95	93.14	92.05	91.15
SVM-Linear	88.03	86.83	88.14	90.82	86.95	90.82	90.69	93.45	92.55	91.74
RF	87.10	87.87	89.65	90.30	88.62	89.21	90.09	91.22	91.97	90.56
KNN	81.59	73.34	75.66	87.24	74.15	86.81	87.03	89.32	90.08	88.01
EMRR	89.53	88.66	93.28	91.12	90.24	**91.06**	**90.75**	**93.86**	**92.62**	**91.91**

#### Effects of intensity interval selection on histogram-based statistical features

**Fig. 9:**
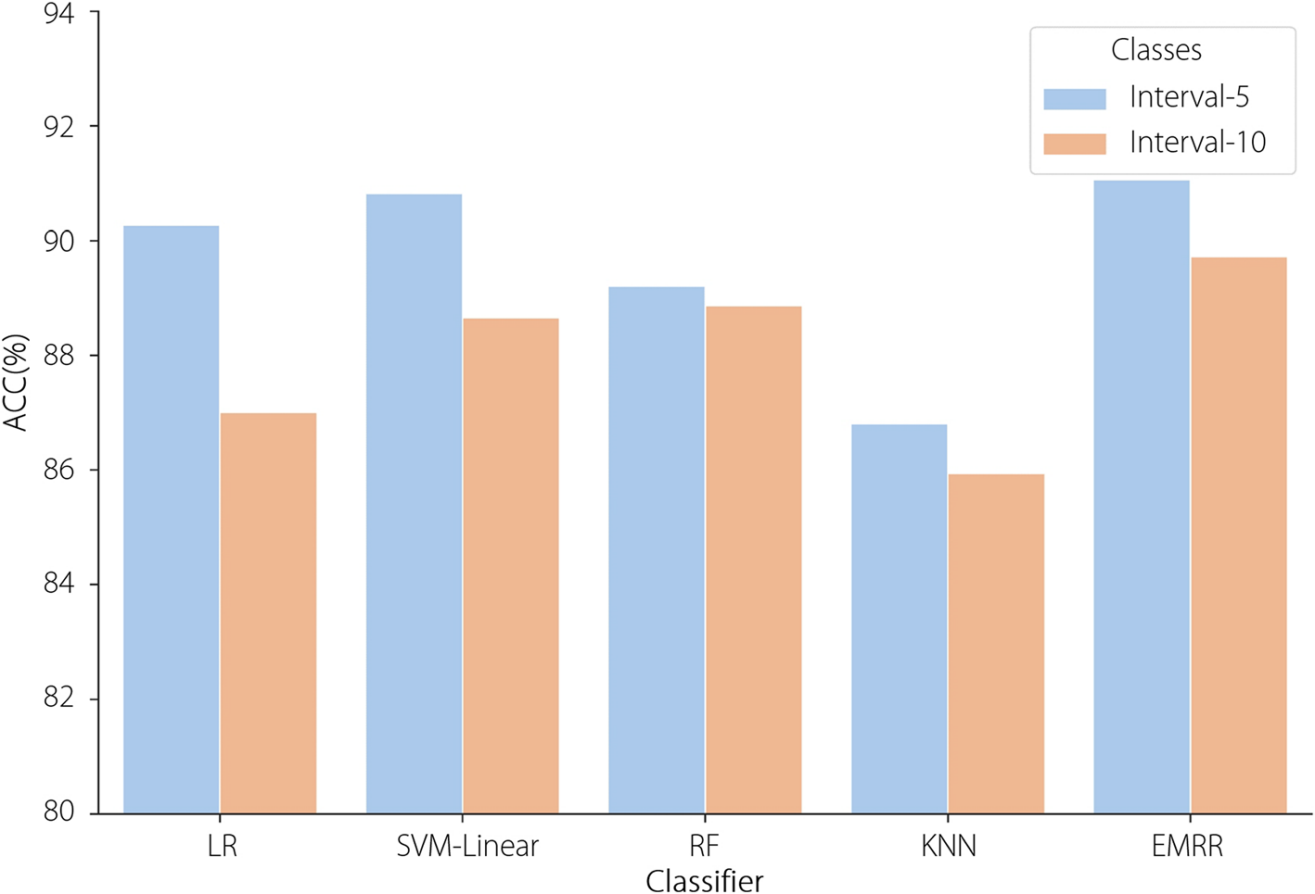
Performance comparison of machine learning methods based on histogram-based statistical features in different intensity intervals

Table 9 and Fig. 9 present the cataracts recognition result comparisons of the histogram-based statistical features based on two intensity interval settings: 10 (interval-10) and 5 (interval-5). The five machine learning methods based on histogram-based statistical features extracted from AS-OCT-based histograms in interval-5 performed better than those in interval-10. The following possible reasons are concluded for this: (1) The features extracted from an intensity interval of 5 are more discriminative than those extracted from an intensity interval of 10. (2) The difference in the intensity distribution between different NC levels is more significant at smaller intervals, which can be easier to recognize.

**Table 9:** Performance comparison of machine learning methods based on histogram-based statistical features in different intensity intervals

Method	Interval-10					Interval-5				
	ACC (%)	SEN (%)	PRE (%)	SPE (%)	F1 (%)	ACC (%)	SEN (%)	PRE (%)	SPE (%)	F1 (%)
LR	87.00	83.72	91.11	89.14	86.41	90.27	89.95	93.14	92.05	91.15
SVM-Linear	88.66	87.36	93.19	90.19	89.20	90.82	90.69	93.45	92.55	91.74
RF	88.87	89.66	90.96	91.69	90.21	89.21	90.09	91.22	91.97	90.56
KNN	85.94	85.53	88.43	89.59	86.84	86.81	87.03	89.32	90.08	88.01
EMRR	89.72	88.61	93.97	91.04	90.33	**91.06**	**90.75**	**93.86**	**92.62**	**91.91**

#### Effects of different feature selection methods

Table 10 shows the cataracts recognition result comparisons of different feature selection methods: original, LDA-based, PCA-based, RFE-based, RFA-based, and the proposed method. The proposed feature selection method selected the best feature subset for cataracts recognition. RFE and RFA obtained better feature subsets, whereas LDA and PCA obtained worse subsets. The results are expained as follows: (1) LDA fails because it projects all features into two dimensions, which may result in the loss of significant feature information. (2) PCA selects only six features as the selection result and ignores the joint contributions of the other features. (3) RFE and RFA are experimental feature selection methods that do not consider the correlations between features, thereby limiting their performance. (4) The proposed method not only analyzes the importance of visual features from global and local perspectives, but also considers the joint contributions of each feature.

**Table 10:** Comparison of recognition results between different feature selection methods

	ACC (%)					
Method	LR	SVM-Linear	RF	KNN	EMRR	Features
Original	90.61	90.72	89.21	86.68	91.30	27
LDA	28.24	27.93	30.83	30.33	26.77	2
PCA	89.64	89.85	89.11	85.47	87.74	6
RFE	91.38	90.59	89.53	88.71	91.46	13
RFA	91.19	90.67	88.77	87.08	91.85	18
Ours	91.56	90.82	90.14	88.00	92.80	22

### Discussion and limitations

Overall, the experimental results demonstrate that the proposed framework has great potential as a computer-aided medical diagnostic tool for clinical cataracts diagnosis, which is explainable and has fewer parameters and lower computational costs than advanced DNNs. The following reasons can explain the cataracts recognition results: (1) Informative visual features from AS-OCT-based histograms and original AS-OCT images were extracted. (2) SHAP and PCC methods were combined to analyze the importance of visual features and select useful visual features. (3) The selected visual features are significantly correlated with the cataracts severity levels. (4) Owing to the large model size, there is considerable redundant information in the DNNs, which affects the recognition results. (5) The DNNs are easily disturbed by the mass of low-intensity pixels, which this framework removes.

However, the proposed framework still has some limitations: First, visual features from AS-OCT-based histograms and original AS-OCT images based on the entire nucleus region were extracted without considering the asymmetric opacity distributions of cataracts in AS-OCT images. Second, the AS-OCT-NC dataset contains only 12,824 images from one center, which has limitations in validating the effectiveness of the framework. Third, the framework has yet to be deployed on ophthalmic equipment for further verification in real-world medical diagnostic practice. To address these limitations, the following solutions are proposed:

- AS-OCT-based histograms and original AS-OCT images will be constructed based on different regions, e.g., whole-, up-, and down-, to investigate the effects of asymmetric opacity distribution of cataracts on the cataract recognition performance.
- Plans are in place to collect more AS-OCT images from different hospitals and areas. Applying the framework to multi-center data will mitigate the problem of data homogeneity.
- Cooperation with medical device companies and hospitals is planned to develop and deploy a computer-aided medical diagnosis tool for cataract diagnosis.

## Conclusions

This study proposed an explainable machine learning framework for automatic cataracts recognition based on visual features extracted from both AS-OCT-based histograms and original AS-OCT images, consisting of three stages: visual feature extraction, feature importance explanation, and recognition. The experimental results on a clinical AS-OCT-NC dataset show that the proposed framework achieved 92.80% ACC and 94.46% SPE, and outperformed competitive machine learning methods and DNNs. In addition, the framework has good interpretability, requires little memory and computational overhead, and can be easily deployed on resource-limited ophthalmic equipment for clinical cataracts diagnosis. Therefore, the framework can be used as a component of existing NC auxiliary diagnosis systems, and the extracted visual features can also be used as a feature subset of the existing system to assist diagnosis.

In the future, plans include improving visual feature extraction methods and incorporating these visual features into the feature representations of DNNs to enhance performance and interpretability. Furthermore, cooperation with medical device companies and hospitals is planned to deploy the framework clinically.

## Data Availability

The data applied in this study is currently not publicly available due to reasons of information sensitivity and are available from the corresponding authors upon reasonable request. All data are located in data storage with controlled access at iMED lab at Southern University of Science and Technology.

## Abbreviations

ACC: Accuracy
AS-OCT: Anterior segment optical coherence tomography
CC: Cortical cataract
CNN: Convolutional neural network
DNN: Deep neural network
DT: Decision tree
EMRR: Ensemble multi-class ridge regression
F1: F1 score
FN: False negative
FP: False positive
GB: Gradient boosting
KNN: K-nearest neighbors
LDA: Linear discriminant analysis
LR: Logistic regression
NB: Naive Bayes
NC: Nuclear cataract
PCA: Principal component analysis
PCC: Pearson correlation coefficient
PRE: Precision
PSC: Posterior subcapsular cataract
RF: Random forest
RFA: Recursive feature augmentation
RFE: Recursive feature elimination
RNN: Recurrent neural network
RR: Ridge regression
SEN: Sensitivity
SGD: Stochastic gradient descent
SHAP: SHapley Additive exPlanations
SOTA: State-of-the-art
SPE: Specificity
SVM: Support vector machine
SVM-Linear: Support vector machine with linear kernel
SVM-RBF: Support vector machine with radial basis function kernel
TN: True negative
TP: True positive
GCANet: Gated channel attention network
RIRNet: Region-based integration-and-recalibration network
